# Monocytes reprogrammed with lentiviral vectors co-expressing GM-CSF, IFN-α2 and antigens for personalized immune therapy of acute leukemia pre- or post-stem cell transplantation

**DOI:** 10.1007/s00262-019-02406-9

**Published:** 2019-10-18

**Authors:** Julia K. Bialek-Waldmann, Michael Heuser, Arnold Ganser, Renata Stripecke

**Affiliations:** 1 Department of Hematology, Hemostasis, Oncology and Stem Cell Transplantation, Hannover Medical School, Carl-Neuberg-Str.1, 30625 Hannover, Germany; 2 Laboratory of Regenerative Immune Therapies Applied, Excellence Cluster REBIRTH and German Centre for Infection Research (DZIF), Partner Site Hannover, Hannover, Germany

**Keywords:** Leukemia, Immune therapy, Lentivirus, WT1, PIVAC 18

## Abstract

Acute myeloid leukemia (AML) is the most common acute leukemia in adults and overall survival remains poor. Chemotherapy is the standard of care for intensive induction therapy. Patients who achieve a complete remission require post-remission therapies to prevent relapse. There is no standard of care for patients with minimal residual disease (MRD), and stem cell transplantation is a salvage therapy. Considering the AML genetic heterogeneity and the leukemia immune-suppressive properties, novel cellular immune therapies to effectively harness immunological responses to prevent relapse are needed. We developed a novel modality of immune therapy consisting of monocytes reprogrammed with lentiviral vectors expressing GM-CSF, IFN-α and antigens. Preclinical studies in humanized mice showed that the reprogrammed monocytes self-differentiated into highly viable induced dendritic cells (iDCs) in vivo which migrated effectively to lymph nodes, producing remarkable effects in the de novo regeneration of T and B cell responses. For the first-in-man clinical trial, the patient’s monocytes will be transduced with an integrase-defective tricistronic lentiviral vector expressing GM-CSF, IFN-α and a truncated WT1 antigen. For transplanted patients, pre-clinical development of iDCs co-expressing cytomegalovirus antigens is ongoing. To simplify the product chain for a de-centralized supply model, we are currently exploring a closed automated system for a short two-day manufacturing of iDCs. A phase I clinical trial study is in preparation for immune therapy of AML patients with MRD. The proposed cell therapy can fill an important gap in the current and foreseeable future immunotherapies of AML.

## AML and the quest for novel immune therapies

Acute myeloid leukemia (AML) is the most common acute leukemia in adults, being diagnosed predominantly in the elderly [1]. The median age in AML at diagnosis ranges from 66 to 71 years (SEER Cancer Statistics Review 1975–2009) and eligibility of patients capable of receiving intensive chemotherapy reduces with age [2]. AML risk stratification is based on the genetic profile of the disease following recommendations from an international expert panel on behalf of the European LeukemiaNet [1]. The somatically acquired genetic changes in AML are very heterogeneous, but they normally affect the mechanisms of self-renewal, proliferation, and differentiation of hematopoietic progenitor cells [3]. Age, comorbidities, health performance status and the type of AML (de novo, therapy-related, secondary after myelodysplastic/myeloproliferative disease) strongly influence the outcome of the malignancy [4, 5].

For patients considered medically fit, the standard of care (SOC) for intensive induction chemotherapy is the combination of anthracycline with cytarabine (‘3 + 7’). The complete remission (CR) rates range between 65–75% in ≤ 60-year adults and 40–60% in > 60-year elderly patients [1]. If intensive chemotherapy is not an option, alternative therapies include low-dose cytarabine and hypomethylating agents (such as decitabine or azacitidine), but the CR rates are lower, between 10 and 20% [6]. The patients with genetically defined AML subgroups show variable responses to intensive or less intensive induction therapies [4]. After induction chemotherapy and CR, post-remission consolidation therapy is recommended to prevent relapse, but this is still a field of debate for frail elderly patients. For younger patients, the genetic and molecular characteristics of the AML are used to guide the type of consolidation therapy, which can span from higher doses of cytarabine to allogeneic HSCT, with a 5-year OS rate of 40–45% [7]. Since AML is a very heterogeneous malignancy, it is very unlikely that all genetic subgroups will respond similarly to the already established or new agents or combination therapies.

One particularly important aspect is when patients with hemato-pathological CR show positivity by sensitive molecular methods for minimal residual disease (MRD), as MRD indicates a high risk of relapse [8]. MRD assessment by qRT-PCR is established for the 40% of AML patients with NPM1 mutations and translocations of RUNX-RUNXT1 and CBFB-MYH11. Wilms tumor protein 1 (WT1) has also been evaluated as MRD marker and showed prognostic value in AML patients [9]. Heuser and colleagues recently developed a new MRD method based on the mutation status of the patients and next-generation sequencing [10]. Using these methods, nearly all patients will have a MRD marker that can be used for sensitive MRD assessment. Despite intensive therapy including induction and consolidation, over 50% of patients with AML will relapse. Treatment of relapsed AML remains a challenge. To date, there is no standard therapy for these relapsed patients [11]. While salvage chemotherapy leads to CR rates of 40–60% if the CR time was 1 year or longer, this rate declines to 10–15% if the CR time was shorter than 1 year [12].

AML patients with positive MRD are principally candidates for allogeneic HSCT. Patients without appropriate donors and patients for whom allogeneic HSCT is out of the question due to co-morbidities or age, currently have no SOC therapy, and these patients are monitored clinically and receive palliative treatment with hypomethylating agents or an investigational compound upon hematologic relapse. A worldwide study of oral azacitidine as a maintenance therapy in patients with CR was performed. Here, patients were included regardless of MRD status. This study is currently in the follow-up phase and the results are not yet known. Maintenance therapy with oral azacitidine is, therefore, a potential therapy; however, this will at best be used for patients without MRD. For patients with positive MRD and/or without a transplant option, there is currently no approved therapy. Immune therapy can have a favorable effect on the leukemia-free survival of patients in this phase as further described below [13].

## WT1-based immune therapies against AML

WT1 is aberrantly overexpressed in leukemia and lymphoma cells. WT1 functions as a zinc-finger transcription factor relevant as a tumor suppressor gene and in cell growth and differentiation. WT1 is commonly expressed in early hematopoietic stem or progenitor cells and is down-regulated upon differentiation and maturation. Unlike other antigens commonly expressed in normal cells (such as CD33 and fms like tyrosine kinase 3 (FLT3)), WT1 is considered to be a leukemia-associated antigen [14]. In the hematopoietic system, housekeeping WT1 gene expression is mostly restricted to early hematopoietic progenitor cells. In contrast, WT1 overexpression is found in blasts of 89% of adult AML patients at diagnosis, with a 1784-fold increase of WT1 transcripts in peripheral blood and 165-fold increase in bone marrow compared to healthy controls as measured in a study of 133 AML patients [15]. Pioneered by the group of Sugiyama et al. in the early nineties and still broadly explored in Japan, quantification of the levels of WT1 transcripts using qRT-PCR showed a correlation between the WT1 mRNA and occurrence of relapse in leukemia patients with MRD after chemotherapy or allogeneic transplantation, and the authors proposed WT1 mRNA expression levels to be a “panleukemic MRD marker” [16, 17]. For AML patients after HSCT, lower detection of WT1 mRNA transcripts correlated significantly with a longer survival (*p* < 0.01) and, conversely, higher detection of WT1 mRNA was associated with relapse and subsequent death of the patients [9]. The WT1 gene overexpression is an additional molecular biomarker, with significant negative impacts on the CR, disease-free-survival and OS in both the favorable and unfavorable group of patients with  internal tandem duplication (ITD) of the FLT3 gene (FLT3^ITD^) [18].

Several clinical centers have performed trials testing vaccination of AML patients with WT1 immunogenic epitopes. This form of immune therapy proved to be feasible, safe, with clinical responses and benefits observed correlating the development of WT1-specific T cell responses with the normalization/reduction of WT1 mRNA levels [19]. Larger randomized clinical trials are planned and underway to objectively quantify the contribution of WT1 peptide vaccination to clinical responses and long-term survival.

Promising results were also obtained in a phase I trial investigating the effect of vaccination with ex vivo cultivated DCs electroporated with WT1 mRNA [20]. More recently, results were obtained in a phase II trial as post-remission treatment in 30 AML patients [13]. WT1 mRNA-electroporated DCs were safe and prevented or delayed relapse in 43% of AML patients in remission after chemotherapy. OS correlated with molecular and WT1-specific CD8^+^ CTL responses. However, these electroporated DCs did not stimulate CD4^+^ T cell responses or antibody responses against WT1, which may have limited the efficacy of the immune therapeutic response. Interactions between DCs and CD4^+^ follicular T cells in the lymph nodes are complex and crucial for initiating cell-mediated and humoral adaptive immune responses [21]. Currently, a WT1 mRNA-transfected DC vaccine combining the production of DCs in 3 days with Toll-like receptor signaling-induced cell maturation is being tested in clinical trials [22]. Studies characterizing the viability and migration of ex vivo generated DCs after intradermal injection in humans showed that a significant percentage of DCs remained at the site of injection and less than 3% of the applied DCs migrated to the local or distal lymph nodes [23]. This may drastically limit the efficacy of ex vivo generated DC vaccines to promote a functional rebound of immune responses and counteract the immune-suppressive environment established by the malignancy.

## Other immune therapies and immune targets for AML

Other experimental immune therapies against AML being considered involve immune checkpoint inhibitors, monoclonal antibodies or adoptive cellular therapies. Blocking immune checkpoints like CTLA-4 or PD-1 for unleashing antitumor immune responses are a promising strategy, as CTLA-4 blockade has led to increased survival in solid cancers like melanoma [24]. Targeting PD-1 is promising as increased expression of its ligand has been linked to resistance of treatment of AML. So far, no clinical study exploring checkpoint inhibitors showed good responses in AML patients, but several studies including PD-1 or CTLA-4 inhibitors are ongoing [25].

The most targeted and studied antigen for monoclonal antibody therapy in AML is CD33, as CD33 is highly expressed on AML blasts. The first approved anti-CD33 antibody–drug conjugate gemtuzumab ozogamicin showed 30% overall response rate in a phase II clinical trial, but had to be withdrawn from the market due to high side effects. Some investigations are currently ongoing regarding anti-leukemic activity of conjugated anti-CD33 monoclonal antibodies and CD33-based immunotoxins [25].

Adoptive cell therapy is a personalized therapy including infusion of ex vivo expanded CTLs, tumor-infiltrating lymphocytes or donor lymphocyte infusion after HSCT. Novel genetically engineered T cells such as CAR- and TCR-engineered T cells pursue the direct retargeting of the T cells to the antigen-bearing target cell. CD19-targeted CAR-T cell therapies are a breakthrough in cancer therapy and similar immune therapies are desired for AML. So far, no CAR therapy is approved for AML, but several CAR-T cell studies targeting among others CD123 and CD33 are ongoing and results are awaited [26].

Other antigens for vaccine-based therapies that have been clinically investigated are Proteinase-1 (PR1), preferentially expressed antigen of melanoma (PRAME) and Receptor for hyaluronic acid-mediated motility (RAHMM). In a phase I/II clinical trial, PR1 peptide vaccine induced specific immunity with clinical response in patients with myeloid malignancies [27]. PRAME and WT1 peptides have been combined in a DC-based vaccination approach and clinical trials are ongoing (clinical trial identifier NCT02405338 and NCT01734304). RAHMM peptide was tested as antigen in a clinical vaccine study, with three out of six patients with myeloid disorders showing clinical response [28]. The best strategy to achieve a successful outcome of a future immunotherapy is likely to be combinations of different antigens as well as immunotherapeutic approaches, like DC-based vaccine plus checkpoint inhibition or adoptive T cell therapies (T cells recognizing leukemia antigens through the TCR or CAR).

## Reverting the leukemia immune-suppressive microenvironment with cytokines

Development of anti-leukemia immune therapies could be potentially hampered by the pro-leukemogenic mechanisms that serve to suppress both antigen-presenting cells and T cells.

One biologically interesting and well-studied example is the FLT3 ligand-independent growth, caused by FLT3^ITD^ mutations in hematopoietic stem cells. Diagnosis samples obtained from AML patients with the FLT3^ITD^ mutations contained ITD^+^ blasts displaying an immature DC phenotype with both myeloid and plasmacytoid features [29, 30]. These aberrant ITD^+^ DC-like cells showed significantly lower HLA-DR expression levels compared with DCs detectable in ITD^−^ AML samples [29]. These ITD^+^ DC-like cells seemed to be arrested in their terminal differentiation and in vitro treatment with CD40L and CpG resulted in only a partial activation for production of IFN-α, TNF-α, and IL-1α [29]. Further, the occurrence of these arrested ITD^+^ DC-like cells (Lin^−^/HLA-DR^+^/CD11c^+^/CD123^+^) was associated with a total lack of *bona fide* terminal DCs (myeloid DCs: BDCA-1^+^ or BDCA-3^+^; plasmacytoid DC: BDCA-2^+^) [30]. Remarkably, even after months of post-induction chemotherapy and remission, the frequencies of DCs and the patterns of cytokine production measured in PBMCs of ITD^+^ AML patients still remained aberrant. For some long-term-monitored patients, PBMC samples collected after remission and secreting high levels of IL-10, TNF-α, IL-6, and IL-1β seemed to predict relapse a few months later. Cell-free supernatants obtained of the diagnostic samples from these patients stimulated allogeneic monocytes to show a pattern of myeloid-derived suppressor cells (MDSCs) by the cytokines secreted (IL-10, TNF-α, IL-6, and IL-1β). Thus, ITD^+^ AML cells seemed to contain dysfunctional antigen-presenting cells and released factors able to convert monocytes into cells secreting cytokines with a pattern of MDSCs. Underscoring these clinical observations, experimental work exploring a FLT3^ITD^ gene knock-in mouse model demonstrated that mice developed a deregulated production of DCs with dysfunctional immunologic characteristics [31]. Due to these immune-suppressive properties of MRD, anti-leukemia vaccines are most likely to promote functional immune responses only when formulated with potent immune boosters designed to reverse immune suppression and exhaustion of T cells, both in the tumor microenvironment and systemically.

We found that expression of GM-CSF in murine and human acute leukemia made them more immunogenic and stimulated anti-leukemia responses, although this did not stimulate their proliferation [32–34]. In fact, other groups showed that GM-CSF reduced the re-plating ability of murine RUNX1-ETO-expressing AML cells, suggesting a possible tumor suppressor role in leukemia [35].

Interferons are glycoproteins secreted by cells in response to viral infections or to biologic inducers and IFN-α2 has been the most broadly evaluated clinically. IFN-α2 has pleiotropic effects in a variety of malignancies such as anti-angiogenic, immuno-regulatory, differentiation-inducing, anti-proliferative, and pro-apoptotic [36]. IFN-α2 promotes a shift in host immunity against tumors from Th2 bias toward Th1, enhancing cell-mediated cytotoxicity and has a role in attracting Th1 lymphocyte traffic to the tumor [36]. Clinical work showed that patients with recurrent CML and AML treated with a combination of donor lymphocyte transfusions with pre-emptive administration of GM-CSF and IFN-α2 reached long-term remissions, indicating the beneficial effect of cytokine stimulation of graft-versus-leukemia reactions. Kolb et al. inferred that these effects were likely to be due to differentiation of leukemia progenitor cells towards DCs in vivo [37].

## Lentiviral vectors as a robust tool for modification of therapeutic cells

For the past two decades, cells engineered with lentiviral vectors (LVs) have emerged and are in continuous development for immune therapy of solid and hematologic malignancies [38]. LVs can infect both replicating and dormant cells, thus offering a robust and versatile tool to permanently genetically modify hematopoietic stem cells (HSCs), T cells, monocytes and DCs [38]. LVs became in the past years a mainstream vector modality for gene modification of CAR-T cell therapy, a breakthrough approach for cancer immune therapy [39]. CD19-CAR-T cells genetically modified with LVs showed a remarkable efficacy for the treatment of patients suffering from relapsed or refractory B cell malignancies. Early standardization of good manufacturing practices (GMP)-compliant protocols for CAR-T cell production with LVs was a pre-requisite for the success of the CD19-CAR-T clinical development [40]. The US Food and Drug Administration (FDA) and the European Medicines Agency (EMA) approval of the first autologous CAR-T cells resulted in a major boost for clinical development of LVs. Large-scale manufacturing of LVs has been established and optimized in several academic centers and in companies using transient transfection methods and development of stable packaging cell lines. From the regulatory perspective, there is a theoretical possibility that occurrence of replication-competent lentivirus or the incidence of insertional mutagenesis could occur due to the LV integration. Thus, the FDA and EMA require extensive analyses to curb these risks. Nonetheless, thus far no adverse effects have been seen in the clinics that could be associated with the properties of LV gene transfer per se. Nonetheless, translational research exploring non-replicating therapeutic cells are applying integrase-defective lentiviral vectors (IDLVs), which remain present in the modified cells as episome, thus further reducing risks of insertional mutagenesis.

## Reprogramming monocytes with multicistronic lentiviral vectors to differentiate into viable DCs

We tested several combinations of cytokines expressed by LVs in DC precursors and showed that combined expression of GM-CSF and IL-4 was sufficient to promote their autonomous differentiation into highly viable human and murine DCs, capable to stimulate potent T cell responses against melanoma [41, 42]. We continued to develop this “next generation of DCs” [43] to build a broad platform of a cell therapy applicable to immune compromised or immune suppressed individuals against cancer or chronic infections. Different tricistronic vectors expressing GM-CSF, IL-4 and antigens for engineering of induced DCs (iDCs) were validated, e.g., co-expressing the tyrosinase related protein-2 as a melanoma antigen [44, 45], expressing the phosphoprotein 65 (pp65) human cytomegalovirus (HCMV) antigen [46, 47] or expressing a truncated WT1 (tWT1) as a leukemia antigen [48]. iDC-expressing tWT1 (iDCtWT1) stimulated T cells in vitro to cause WT1-dependent cytotoxicity against both autologous and HLA-matched leukemia cells, and promoted large expansion of human T cells in vivo, in immune-deficient mice [48].

Later, mature iDCs expressing pp65 (iDCpp65) with superior T cell stimulatory capabilities in vitro and in vivo were obtained after replacement of IL-4 with IFN-α2 expression [49, 50]. For evaluation of the pharmacodynamic activities of iDCs co-expressing GM-CSF and IFN-α2 in immune-deficient mice transplanted with human HSCs (“humanized mice” recapitulating human HSCT), we explored pp65 as a potent T cell model antigen. Remarkably, s.c. administration of iDCpp65 into humanized mice starting 6 weeks after HSCT stimulated de novo thymic and peripheral CD4^+^ and CD8^+^ development of naive and memory T cells, boosted B cell development, and produced pp65-specific cellular and humoral (IgM and IgG) responses [49, 51]. We developed the standard operating procedures for a swift GMP-compliant iDCpp65 manufacturing and cryopreservation, requiring 2 days of manufacturing, filling and cryopreservation. Quality control of the thawed iDCpp65 product by multicolor flow cytometry and PCR demonstrated high recovery and consistency [50]. Pilot safety studies of iDCpp65 in fully humanized mice maintained for 20–26 weeks after immunization showed no iDC therapy-related tumorigenic or toxicity effects [52, 53].

## Clinical development of iDCtWT1 co-expressing GM-CSF, IFN-α2 and tWT1

**Fig. 1:**
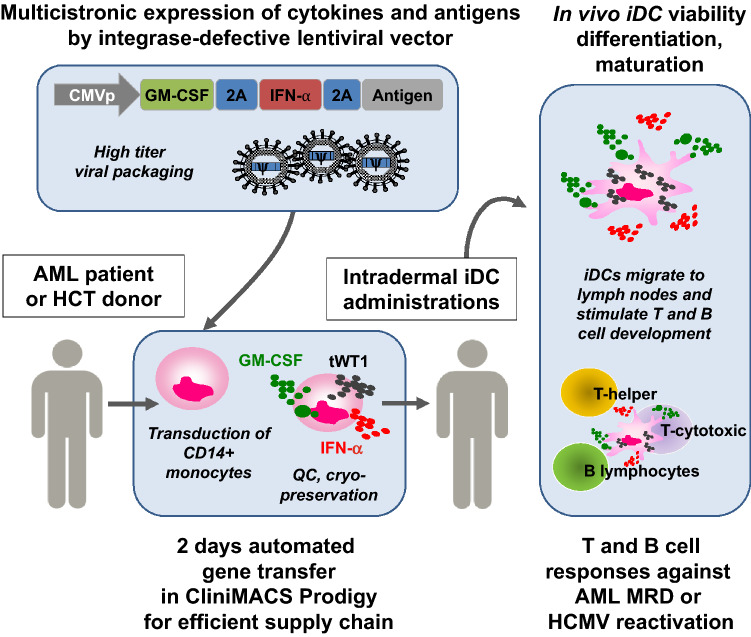
Schematic representation of the tricistronic integrase-defective lentiviral vector design and manufacturing process of reprogrammed monocytes that self-differentiate into induced dendritic cells (iDC). CD14^+^ monocytes obtained from the AML patient or HSCT donor are enriched and transduced with high titer lentiviral vector in a CliniMACS Prodigy automated system. After quality control, the cryopreserved iDC cell product can be stored or shipped to other clinical centers. After thawing of the product and intradermal administration into patients, highly viable self-differentiated iDC will secrete GM-CSF and IFN-α2 and process antigens, stimulating the development of functional T and B cell responses. The combined adaptive responses will protect patients against AML relapse (tWT1 antigen) or HCMV reactivation (HCMV antigens pp65/gB)

More recently, iDCtWT1 co-expressing GM-CSF, IFN-α2 and tWT1 were developed and validated pre-clinically using monocytes from adult (peripheral blood or leukapheresis) and neonate (cord-blood) donors (Bialek-Waldmann et al., manuscript in preparation). For a clinical trial, adult MDR^+^ AML patients will receive the iDCtWT1 “drug product” defined as the cryopreserved lentivirus-transduced CD14^+^ cells in the final formulation (Fig. 1). In the European Union, the cell product will be classified as an advanced therapeutic medicinal product (ATMP). Since LVs are used for production of iDCs, they will be further classified as gene therapy medicinal product (GTMP) and, as such, will be subjected to particular regulatory regulations, especially considering safety requirements. Due to their high performance, safety profile (no occurrence of replication competent lentivirus has been reported) and extensive clinical use (particularly with the advent of an approved CAR-T cell product CTL019 or Kymriah) LVs are currently the preferred viral vector type for gene therapy clinical trials. Since DCs do not replicate, we chose to use IDLVs to further enhance safety by minimizing the chances of insertional mutagenesis to occur. The expected pharmacological effects of the product are: (1) In vivo self-differentiation of monocytes into highly viable iDCtWT1, (2) Effective bio-distribution and homing in adjacent and distal lymph nodes, (3) Presentation of several WT1 class I and class II epitopes to CD8^+^ and CD4^+^ T cells; (4) Through secretion of GM-CSF and IFN-α2, autocrine and paracrine effects will abrogate the immune suppression and promote stimulation of de novo T cell and B cell responses. The first-in-man autologous iDCtWT1 cell vaccine reprogrammed ex vivo after a short automated manipulation with a tricistronic self-inactivating IDLV will be evaluated in a Phase I/IIa multicentric trial as immune therapy of adult MDR^+^ AML patients.

## Automated production of iDCs

We have previously shown feasibility of GMP-compliant generation of iDCs with leukapheresis of healthy donors using a CliniMACS immunomagnetic separation system (Miltenyi Biotec) for isolation of monocytes, transfer into culture bags and cultivation and transduction in bags [45, 50]. However, major logistics limitations for a decentralized iDC supply are the need for high-level clean-room facilities, related infrastructure and training of the personnel for the manufacturing. To reduce the hands-on requirements and need of clean-room resources, an automated closed manufacturing system is currently being used to generate iDCs under GMP-compliant conditions (Bialek-Waldmann et al., manuscript in preparation). The CliniMACS Prodigy (Miltenyi Biotec, Germany) is an integrated clinical-scale manufacturing system for cellular products which enables the full manufacturing process of cellular products including magnetic cell separation, transduction and cultivation in a GMP-compliant single-use tubing set [54]. According to the GMP for ATMP guideline of the European Commission, operation of closed systems will require cleanrooms of class D or higher. Operation in cleanrooms of class C or D would reduce the requirements for space and standards (such as number of particles in the air) compared to manual manufacturing. Processes using automated manufacturing system were recently successfully implemented for manufacturing several cell products [55–60]. The ex vivo generation of monocyte-derived DCs in CliniMACS Prodigy has first been described by Erdmann and colleagues [61]. Production in this study was feasible and yields were comparable to non-automated standard protocol (dendritic cell recovery of 23% and 26%, respectively) [61]. For ethical reasons, we initiated the process development using leukapheresis material obtained from healthy donors and for future validation runs, we will include leukapheresis from AML patients as well. For manufacturing of first-in-man autologous iDCtWT1 cell vaccine, we chose to adapt CliniMACS Prodigy to enrich CD14^+^ monocytes using CD14 MicroBeads and to cultivate and transduce CD14^+^ monocytes in the CentriCult chamber. After enrichment of CD14^+^ monocytes, cells were cultured in the presence of recombinant GM-CSF (to activate monocytes) and IL-4 (to inhibit macrophage differentiation) and transduced with tricistronic IDLV expressing GM-CSF, IFN-α2 and a tWT1. The process includes in-process monitoring of the starting material (leukapheresis), after CD14 enrichment and after transduction, determining cell yield (cell counts), purity and viability of the cell product (flow cytometry) and detection of lentiviral sequences by qRT-PCR in total DNA of the iDCtWT1 product (Bialek-Waldmann et al. manuscript in preparation). For pilot studies, isolation of CD14^+^ monocytes from leukapheresis, cultivation and transduction in CliniMACS Prodigy was feasible in a two-day process, paving the way for completing the GMP-compliant automated manufacturing process of iDCtWT1 for future multicenter clinical trials.

## Conclusion and outlook

Highly viable iDCs engineered with LVs for secretion of GM-CSF and IFN-α2 can stimulate the de novo generation of long-lasting T and B cell responses. As modular technology based on a simple combinatorial gene transfer and simple and fast ex vivo manipulation, the applications are broad in the field of immune therapy against cancer and chronic viral infections. The unique features of this novel modality cell vaccine will be explored to treat AML patients against leukemia relapse and patients after HSCT against HCMV viral reactivations. Cryopreservation and currently ongoing strategies for automated manufacturing of iDCs will allow a decentralized supply chain for distribution of the iDC products to several clinical centers. Our hypothesis for the first-in-man clinical trial is that iDCtWT1 will revert the immune suppression impinged by AML MRD and will eventually protect patients long term against the leukemia relapse.

## Abbreviations

AML: Acute myeloid leukemia
ATMP: Advanced therapeutic medicinal product
CR: Complete remission
EMA: European Medicines Agency
FDA: US Food and Drug Administration
FLT3: Fms like tyrosine kinase 3
FLT3^ITD^: Internal tandem duplication of FLT3
GMP: Good manufacturing practices
HCMV: Human cytomegalovirus
iDC: Induced dendritic cell
iDCpp65: iDC expressing pp65
iDCtWT1: iDC expressing tWT1
IDLV: Integrase-defective lentiviral vectors
ITD: Internal tandem duplication
LV: Lentiviral vectors
MDSC: Myeloid-derived suppressor cell
MRD: Minimal residual disease
pp65: Phosphoprotein 65
PR1: Proteinase-1
PRAME: Preferentially expressed antigen of melanoma
RAHMM: Receptor for hyaluronic acid-mediated motility
SOC: Standard of care
tWT1: Truncated WT1
WT1: Wilms tumor protein 1

## References

[1] Dohner H, Estey E, Grimwade D (2017). Diagnosis and management of AML in adults: 2017 ELN recommendations from an international expert panel. Blood.

[2] Juliusson G, Antunovic P, Derolf A, Lehmann S, Mollgard L, Stockelberg D, Tidefelt U, Wahlin A, Hoglund M (2009). Age and acute myeloid leukemia: real world data on decision to treat and outcomes from the Swedish Acute Leukemia Registry. Blood.

[3] TCGA (2013). Genomic and epigenomic landscapes of adult de novo acute myeloid leukemia. N Engl J Med.

[4] Dohner H, Estey EH, Amadori S (2010). Diagnosis and management of acute myeloid leukemia in adults: recommendations from an international expert panel, on behalf of the European LeukemiaNet. Blood.

[5] Gerstung M, Papaemmanuil E, Martincorena I (2017). Precision oncology for acute myeloid leukemia using a knowledge bank approach. Nat Genet.

[6] Dombret H, Seymour JF, Butrym A (2015). International phase 3 study of azacitidine vs conventional care regimens in older patients with newly diagnosed AML with > 30% blasts. Blood.

[7] Burnett A, Wetzler M, Lowenberg B (2011). Therapeutic advances in acute myeloid leukemia. J Clin Oncol.

[8] Ivey A, Hills RK, Simpson MA (2016). Assessment of minimal residual disease in standard-risk AML. N Engl J Med.

[9] Casalegno-Garduno R, Schmitt A, Spitschak A (2016). Immune responses to WT1 in patients with AML or MDS after chemotherapy and allogeneic stem cell transplantation. Int J Cancer.

[10] Thol F, Gabdoulline R, Liebich A (2018). Measurable residual disease monitoring by NGS before allogeneic hematopoietic cell transplantation in AML. Blood.

[11] Thol F, Schlenk RF, Heuser M, Ganser A (2015). How I treat refractory and early relapsed acute myeloid leukemia. Blood.

[12] Estey E, Kornblau S, Pierce S, Kantarjian H, Beran M, Keating M (1996). A stratification system for evaluating and selecting therapies in patients with relapsed or primary refractory acute myelogenous leukemia. Blood.

[13] Anguille S, Van de Velde AL, Smits EL (2017). Dendritic cell vaccination as postremission treatment to prevent or delay relapse in acute myeloid leukemia. Blood.

[14] Lyu X, Xin Y, Mi R (2014). Overexpression of Wilms tumor 1 gene as a negative prognostic indicator in acute myeloid leukemia. PLoS ONE.

[15] Ostergaard M, Olesen LH, Hasle H, Kjeldsen E, Hokland P (2004). WT1 gene expression: an excellent tool for monitoring minimal residual disease in 70% of acute myeloid leukaemia patients - results from a single-centre study. Br J Haematol.

[16] Inoue K, Sugiyama H, Ogawa H (1994). WT1 as a new prognostic factor and a new marker for the detection of minimal residual disease in acute leukemia. Blood.

[17] Ogawa H, Tamaki H, Ikegame K (2003). The usefulness of monitoring WT1 gene transcripts for the prediction and management of relapse following allogeneic stem cell transplantation in acute type leukemia. Blood.

[18] Damm F, Heuser M, Morgan M (2011). Integrative prognostic risk score in acute myeloid leukemia with normal karyotype. Blood.

[19] Di Stasi A, Jimenez AM, Minagawa K, Al-Obaidi M, Rezvani K (2015). Review of the results of WT1 peptide vaccination strategies for myelodysplastic syndromes and acute myeloid leukemia from nine different studies. Front Immunol.

[20] Van Tendeloo VF, Van de Velde A, Van Driessche A (2010). Induction of complete and molecular remissions in acute myeloid leukemia by Wilms’ tumor 1 antigen-targeted dendritic cell vaccination. Proc Natl Acad Sci USA.

[21] Bousso P (2008). T-cell activation by dendritic cells in the lymph node: lessons from the movies. Nat Rev Immunol.

[22] Subklewe M, Geiger C, Lichtenegger FS, Javorovic M, Kvalheim G, Schendel DJ, Bigalke I (2014). New generation dendritic cell vaccine for immunotherapy of acute myeloid leukemia. Cancer Immunol Immunother.

[23] De Vries IJ, Krooshoop DJ, Scharenborg NM (2003). Effective migration of antigen-pulsed dendritic cells to lymph nodes in melanoma patients is determined by their maturation state. Cancer Res.

[24] Hodi FS, O’Day SJ, McDermott DF (2010). Improved survival with ipilimumab in patients with metastatic melanoma. N Engl J Med.

[25] Masarova L, Kantarjian H, Garcia-Mannero G, Ravandi F, Sharma P, Daver N (2017). Harnessing the immune system against leukemia: monoclonal antibodies and checkpoint strategies for AML. Adv Exp Med Biol.

[26] Hofmann S, Schubert ML, Wang L, He B, Neuber B, Dreger P, Muller-Tidow C, Schmitt M (2019). Chimeric antigen receptor (CAR) T cell therapy in acute myeloid leukemia (AML). J Clin Med..

[27] Qazilbash MH, Wieder E, Thall PF (2017). PR1 peptide vaccine induces specific immunity with clinical responses in myeloid malignancies. Leukemia.

[28] Schmitt M, Schmitt A, Rojewski MT (2008). RHAMM-R3 peptide vaccination in patients with acute myeloid leukemia, myelodysplastic syndrome, and multiple myeloma elicits immunologic and clinical responses. Blood.

[29] Rickmann M, Krauter J, Stamer K, Heuser M, Salguero G, Mischak-Weissinger E, Ganser A, Stripecke R (2011). Elevated frequencies of leukemic myeloid and plasmacytoid dendritic cells in acute myeloid leukemia with the FLT3 internal tandem duplication. Ann Hematol.

[30] Rickmann M, Macke L, Sundarasetty BS (2013). Monitoring dendritic cell and cytokine biomarkers during remission prior to relapse in patients with FLT3-ITD acute myeloid leukemia. Ann Hematol.

[31] Lau CM, Nish SA, Yogev N, Waisman A, Reiner SL, Reizis B (2016). Leukemia-associated activating mutation of Flt3 expands dendritic cells and alters T cell responses. J Exp Med.

[32] Stripecke R, Skelton DC, Gruber T, Afar D, Pattengale PK, Witte ON, Kohn DB (1998). Immune response to Philadelphia chromosome-positive acute lymphoblastic leukemia induced by expression of CD80, interleukin 2, and granulocyte-macrophage colony-stimulating factor. Hum Gene Ther.

[33] Stripecke R, Cardoso AA, Pepper KA, Skelton DC, Yu XJ, Mascarenhas L, Weinberg KI, Nadler LM, Kohn DB (2000). Lentiviral vectors for efficient delivery of CD80 and granulocyte-macrophage- colony-stimulating factor in human acute lymphoblastic leukemia and acute myeloid leukemia cells to induce antileukemic immune responses. Blood.

[34] Koya RC, Kasahara N, Pullarkat V, Levine AM, Stripecke R (2002). Transduction of acute myeloid leukemia cells with third generation self-inactivating lentiviral vectors expressing CD80 and GM-CSF: effects on proliferation, differentiation, and stimulation of allogeneic and autologous anti-leukemia immune responses. Leukemia.

[35] Matsuura S, Yan M, Lo MC (2012). Negative effects of GM-CSF signaling in a murine model of t(8;21)-induced leukemia. Blood.

[36] Rafique I, Kirkwood JM, Tarhini AA (2015). Immune checkpoint blockade and interferon-alpha in melanoma. Semin Oncol.

[37] Kolb HJ, Rank A, Chen X, Woiciechowsky A, Roskrow M, Schmid C, Tischer J, Ledderose G (2004). In-vivo generation of leukaemia-derived dendritic cells. Best Pract Res Clin Haematol.

[38] Olbrich H, Slabik C, Stripecke R (2017). Reconstructing the immune system with lentiviral vectors. Virus Genes.

[39] Porter DL, Kalos M, Zheng Z, Levine B, June C (2011). Chimeric antigen receptor therapy for B-cell malignancies. J Cancer..

[40] Gill S, Maus MV, Porter DL (2015). Chimeric antigen receptor T cell therapy: 25 years in the making. Blood Rev.

[41] Koya RC, Weber JS, Kasahara N, Lau R, Villacres MC, Levine AM, Stripecke R (2004). Making dendritic cells from the inside out: lentiviral vector-mediated gene delivery of granulocyte-macrophage colony-stimulating factor and interleukin 4 into CD14 + monocytes generates dendritic cells in vitro. Hum Gene Ther.

[42] Koya RC, Kimura T, Ribas A (2007). Lentiviral vector-mediated autonomous differentiation of mouse bone marrow cells into immunologically potent dendritic cell vaccines. Mol Ther.

[43] Carroll RG, June CH (2007). Programming the next generation of dendritic cells. Mol Ther.

[44] Pincha M, Sundarasetty BS, Salguero G (2012). Identity, potency, in vivo viability, and scaling up production of lentiviral vector-induced dendritic cells for melanoma immunotherapy. Hum Gene Ther Methods.

[45] Sundarasetty BS, Chan L, Darling D (2015). Lentivirus-induced ‘Smart’ dendritic cells: Pharmacodynamics and GMP-compliant production for immunotherapy against TRP2-positive melanoma. Gene Ther.

[46] Salguero G, Sundarasetty BS, Borchers S (2011). Preconditioning therapy with lentiviral vector-programmed dendritic cells accelerates the homeostatic expansion of antigen-reactive human T cells in NOD.Rag1-/-.IL-2rgammac-/- mice. Hum Gene Ther.

[47] Daenthanasanmak A, Salguero G, Borchers S (2012). Integrase-defective lentiviral vectors encoding cytokines induce differentiation of human dendritic cells and stimulate multivalent immune responses in vitro and in vivo. Vaccine.

[48] Sundarasetty BS, Singh VK, Salguero G (2013). Lentivirus-induced dendritic cells for immunization against high-risk WT1(+) acute myeloid leukemia. Hum Gene Ther.

[49] Daenthanasanmak A, Salguero G, Sundarasetty BS (2015). Engineered dendritic cells from cord blood and adult blood accelerate effector T cell immune reconstitution against HCMV. Mol Ther Methods Clin Dev.

[50] Sundarasetty BS, Kloess S, Oberschmidt O (2015). Generation of lentivirus-induced dendritic cells under GMP-compliant conditions for adaptive immune reconstitution against cytomegalovirus after stem cell transplantation. J Transl Med.

[51] Salguero G, Daenthanasanmak A, Munz C (2014). Dendritic cell-mediated immune humanization of mice: implications for allogeneic and xenogeneic stem cell transplantation. J Immunol.

[52] Volk V, Reppas AI, Robert PA (2017). Multidimensional analysis integrating human T-cell signatures in lymphatic tissues with sex of humanized mice for prediction of responses after dendritic cell immunization. Front Immunol.

[53] Sundarasetty BS, Volk V, Theobald SJ (2017). Human effector memory T helper cells engage with mouse macrophages and cause graft-versus-host-like pathology in skin of humanized mice used in a nonclinical immunization study. Am J Pathol.

[54] Apel M, Brüning M, Granzin M (2013). Integrated clinical scale manufacturing system for cellular products derived by magnetic cell separation, centrifugation and cell culture. Chem Ing Tec.

[55] Kumaresan P, Figliola M, Moyes JS, Huls MH, Tewari P, Shpall EJ, Champlin R, Cooper LJ (2015). Automated cell enrichment of cytomegalovirus-specific T cells for clinical applications using the cytokine-capture system. J Vis Exp.

[56] Pello OM, Innes AJ, Bradshaw A, Finn SA, Uddin S, Bray E, Olavarria E, Apperley JF, Pavlu J (2017). BKV-specific T cells in the treatment of severe refractory haemorrhagic cystitis after HLA-haploidentical haematopoietic cell transplantation. Eur J Haematol.

[57] Priesner C, Aleksandrova K, Esser R (2016). Automated enrichment, transduction, and expansion of clinical-scale CD62L(+) T cells for manufacturing of gene therapy medicinal products. Hum Gene Ther.

[58] Granzin M, Soltenborn S, Muller S, Kollet J, Berg M, Cerwenka A, Childs RW, Huppert V (2015). Fully automated expansion and activation of clinical-grade natural killer cells for adoptive immunotherapy. Cytotherapy.

[59] Kloss S, Oberschmidt O, Morgan M (2017). Optimization of human NK cell manufacturing: fully automated separation, improved ex vivo expansion using IL-21 with autologous feeder cells, and generation of anti-CD123-CAR-expressing effector cells. Hum Gene Ther.

[60] Roddie C, O’Reilly M, Dias Alves Pinto J, Vispute K, Lowdell M (2019). Manufacturing chimeric antigen receptor T cells: issues and challenges. Cytotherapy.

[61] Erdmann M, Uslu U, Wiesinger M, Bruning M, Altmann T, Strasser E, Schuler G, Schuler-Thurner B (2018). Automated closed-system manufacturing of human monocyte-derived dendritic cells for cancer immunotherapy. J Immunol Methods.

